# A novel prognostic model for ovarian cancer: construction, validation, and therapeutic insights

**DOI:** 10.3389/fgene.2026.1603788

**Published:** 2026-04-22

**Authors:** Tingting Yu, Mingyang Gao, Yuexin Yu, Bolun Wang

**Affiliations:** Department of Reproductive Medicine, General Hospital of Northern Theater Command, Shenyang, Liaoning, China

**Keywords:** drug sensitivity, immunotherapy, ovarian cancer, palmitoylation, prognostic model

## Abstract

**Background:**

Ovarian cancer (OV) is the most lethal gynaecological malignancy worldwide. Palmitoylation, a reversible post-translational lipid modification, has been implicated in tumourigenesis, growth, metastasis and apoptosis across multiple cancers. However, its impact on immune infiltration, therapeutic response and clinical outcomes in ovarian cancer remains insufficiently explored.

**Methods:**

We obtained transcriptome data and clinical information pertaining to ovarian cancer from the Cancer Genome Atlas (TCGA), Genotype-Tissue Expression (GTEx), and Gene Expression Omnibus (GEO) databases. A prognostic model based on palmitoylation-related genes was constructed using univariate Cox and Lasso-Cox regression for feature selection. The predictive performance of the model was assessed via Kaplan-Meier (KM) survival analysis and receiver operating characteristic (ROC) curve evaluation.

**Results:**

We developed a five-gene prognostic prediction model utilizing palmitoylation-related genes derived from TCGA samples of epithelial ovarian cancer patients. The validity of this model was confirmed using patient samples from both TCGA and GEO datasets. KM analysis demonstrated that our prognostic model effectively distinguished between high-risk and low-risk groups, correlating with poorer or more favorable outcomes respectively. According to ROC curve analysis, our model exhibited superior predictive accuracy compared to traditional clinical factors alone. Additionally, analyses regarding immune cell infiltration, expression levels of immune checkpoints, as well as drug sensitivity further support potential treatment strategies for ovarian cancer.

**Conclusion:**

The prognostic model developed in this study has the potential to enhance our understanding of the role of palmitic acid-related genes in ovarian cancer, providing new insights into prognosis prediction and treatment strategies for patients with ovarian cancer.

## 1 Introduction

Ovarian cancer (OV) is one of the most aggressive and lethal gynecological malignancies, characterized by a poor prognosis that results in over 207,252 cancer-related deaths globally each year (Webb and Jordan, 2024). While surgical resection combined with adjuvant chemotherapy can improve long-term survival rates for patients with resectable disease, most individuals are diagnosed at advanced stages where treatment options become limited. Despite advancements in surgical techniques and chemotherapy regimens over recent years, the overall 5-year survival rate for patients with advanced OV remains dismally low, typically below 50% (Pfisterer et al., 2020). Consequently, the molecular heterogeneity inherent to this disease underscores the urgent need for reliable biomarkers that can enhance prognostic accuracy and inform personalized treatment approaches.

Palmitoylation is a reversible post-translational lipid modification involving the addition of a 16-carbon palmitoyl group to cysteine residues on proteins via thioester bonds (Li et al., 2025). S-palmitoylation is catalyzed by palmitoyl S-acyltransferases (PATs), which contain the essential zinc-finger Asp-His-His-Cys (DHHC) domain required for transferring palmitic acid to target proteins (Wang et al., 2025). This modification plays a critical role in regulating protein localization, accumulation, secretion, stability, and functional integrity (Chen et al., 2017). Disruption of palmitoylation can interfere with cellular pathways and contribute to various diseases—particularly cancers. Extensive research has demonstrated that aberrant palmitoylation is associated with tumorigenesis as well as growth metastasis and apoptosis (Lothrop et al., 2013; Zhang et al., 2021). Recent studies indicate that S-palmitoylation of proteins within tumor or immune cells influences the tumor immune microenvironment by modulating immune cell activation, depletion, and infiltration (Yao et al., 2019). For instance, ZDHHC9-mediated palmitoylation plays a crucial role in regulating the stability of programmed cell death 1 ligand 1 (PD-L1), which interacts with programmed cell death 1 (PD-1) on T cells to transmit immunosuppressive signals (Zou et al., 2016). Notably, the inhibition of PD-L1 palmitoylation—achieved through mutation of the palmitoylation site or knockdown of palmitoylases using shRNA—sensitized breast cancer cell lines to T-cell-mediated cytotoxicity, thereby impeding tumor growth in murine models (Yang et al., 2019). Furthermore, ZDHHC3-mediated palmitoylation of B7-H4 has been shown to prevent lysosomal degradation and suppress T-cell activation in high-grade serous ovarian cancer, thereby maintaining tumor immune evasion (Yan et al., 2025). Despite the fact that palmitoylation-related genes have been implicated in tumor progression, immune evasion, and therapeutic responses (Feng et al., 2024), their functional mechanisms in OV remain insufficiently characterised. In particular, there is a paucity of research into the unique roles of these cells in shaping the OV immune microenvironment, as well as their impacts on immune infiltration, treatment efficacy, and clinical prognosis. Consequently, this research aims to elucidate the mechanisms by which palmitoylation-related genes operate in OV and to develop prognostic models that could offer new insights into ovarian cancer treatment and enhance patient prognosis (Li et al., 2021).

## 2 Materials and methods

### 2.1 Differential gene expression analysis

Transcriptome data for TCGA ovarian cancer (OV) and GTEx normal ovarian tissues were downloaded from the UCSC Xena Browser (https://xenabrowser.net/). Samples with essential clinical information were retained, yielding a final cohort of 415 tumor tissues and 88 normal tissues. Expression data were uniformly converted to log2 (TPM+1) scale. The R package “Limma” v3.60.6 was utilized to analyze the differentially expressed genes in the log_2_ (TPM+1) data of ovarian tumor and normal tissues (|log_2_FC| > 1, p < 0.05). The ggplot2 package was employed to create a volcano plot illustrating the differential gene expression.

### 2.2 Construction and validation of prognostic models

A total of 415 TCGA cases were randomly divided into training and internal validation sets at an 8:2 ratio, with model construction performed using the training set.palmitoylation-related protein-coding genes (n = 3,310) were retrieved from the MSigDB database (https://www.gsea-msigdb.org/) and SwissPalm database, which intersected with differentially expressed genes (DEGs), yielding 1,379 overlapping genes. Subsequently, univariate Cox analysis was performed using the R package “survival” v3.7.0, and genes with significant prognostic correlation (p < 0.01) were selected. The least absolute shrinkage and selection operator (LASSO)-penalized Cox regression analysis was employed to refine the candidate gene for prognostic model development using the R package “glmnet”. The penalty parameter (λ) was determined via ten-fold cross-validation by selecting the value that met the minimum criterion. Ultimately, five genes were identified to construct the prognostic signature. The risk score model was established by multiplying the coefficient (β) value with the expression level of each risk gene, formulated as follows: risk score = MYL2 × 0.9724 + CACNA1C × 0.3278 + WASF2 × 0.1686 + GRB7 × 0.208 − CXCL13 × 0.1425.

Survival and clinical information from patients in the TCGA and GEO datasets were obtained. Patients were stratified into high-risk and low-risk groups based on the median risk score, and a comparison of overall survival rates between these groups was conducted, as illustrated by Kaplan-Meier (KM) curves. The effectiveness of the model was evaluated through the R packages “survminer” v0.4.9, and “timeROC” v0.4 by performing receiver operating characteristic (ROC) curves and calculating the Area Under the Curve (AUC) values. Additionally, The performance of the prognostic model was evaluated using GSE102073 as an external test set.

### 2.3 Analysis of clinical features

Candidate clinical factors (including patient age, clinical stage, risk score and tumor grade) from the TCGA dataset were screened using univariate Cox regression analysis, and variables with p < 0.05 were retained. A nomogram was developed using multivariate Cox regression based on the retained clinical variables. The predictive accuracy of the clinical model was evaluated by calculating the C-index and constructing calibration curves in both the training and internal validation cohorts.

### 2.4 Construction of similar gene networks

To identify genes associated with prognostic markers, GeneMANIA (https://genemania.org/) was used to map interaction networks involving these prognostic genes.

### 2.5 Somatic mutation analysis

To investigate the impact of gene mutations on prognosis, data from TCGA was analyzed using the GSCA “TCGAmutations” database (https://guolab.wchscu.cn/GSCA) along with the R package “maftools” for the identification of somatic mutations.

### 2.6 Drug sensitivity analysis

Patients in the TCGA were categorized into high-risk and low-risk groups based on their median risk scores. The R package “OncoPredict” was utilized to predict IC_50_ values for 546 drugs across both risk categories, and box plots illustrating these findings were generated using ggplot2 for presentation purposes. Additionally, correlations between the drugs and prognostic genes were calculated using Pearson correlation method.

### 2.7 Analysis of immune infiltration and immune scores

The R packages “CIBERSORT” v0.1.0 and “estimate” v1.0.13 were used to assess the extent of immune cell infiltration and to calculate the immune score in TCGA samples, respectively. The rank-sum test was used to evaluate the differences in the abundance of immune infiltration and immune scores of 22 immune cell types between the high-risk and low-risk patient groups.

### 2.8 Single-cell data processing

The single-cell RNA-seq dataset GSE184880, comprising five normal and seven ovarian cancer tissues, was downloaded. Data processing was performed using the Seurat R package (v5.1.0). Quality control (QC) metrics were applied to exclude low-quality cells based on the following criteria: nFeature_RNA between 500 and 6,000, nCount_RNA between 200 and 30,000, and percentage of mitochondrial gene expression (per.mt) < 20%. To mitigate ambient RNA contamination, cell-level contamination was estimated using decontX (v1.4.0) with a threshold of 0.2 for filtering. Additionally, to detect and remove potential doublets, cells were further filtered based on the classification results from scDblFinder (v1.18.0), which computes doublet probability scores from the count matrix. The number of reduced-dimension principal components was set to 12, and the clustering resolution was selected as 0.3.

### 2.9 Cell clustering and cell annotation

The standard Seurat pipeline was then utilised to perform data normalisation, identify highly variable genes, conduct principal component analysis (PCA), and perform unsupervised clustering. Harmony v1.2.0 was employed for batch correction and data integration, with the sample identity specified as the batch variable. The RunHarmony () function was executed with default parameters, retaining the first 12 principal components for downstream analysis. The clustering resolution was set at 0.3, and UMAP was employed for the purposes of dimensionality reduction and visualisation. The annotation of cell types was conducted using marker genes. The R package CellChat v1.6.1 was used to analyze the cell communication among the nine cell types annotated in the single-cell dataset GSE184880.

## 3 Results

### 3.1 Identification of palmitoylation-related genes in ovarian cancer

**FIGURE 1:**
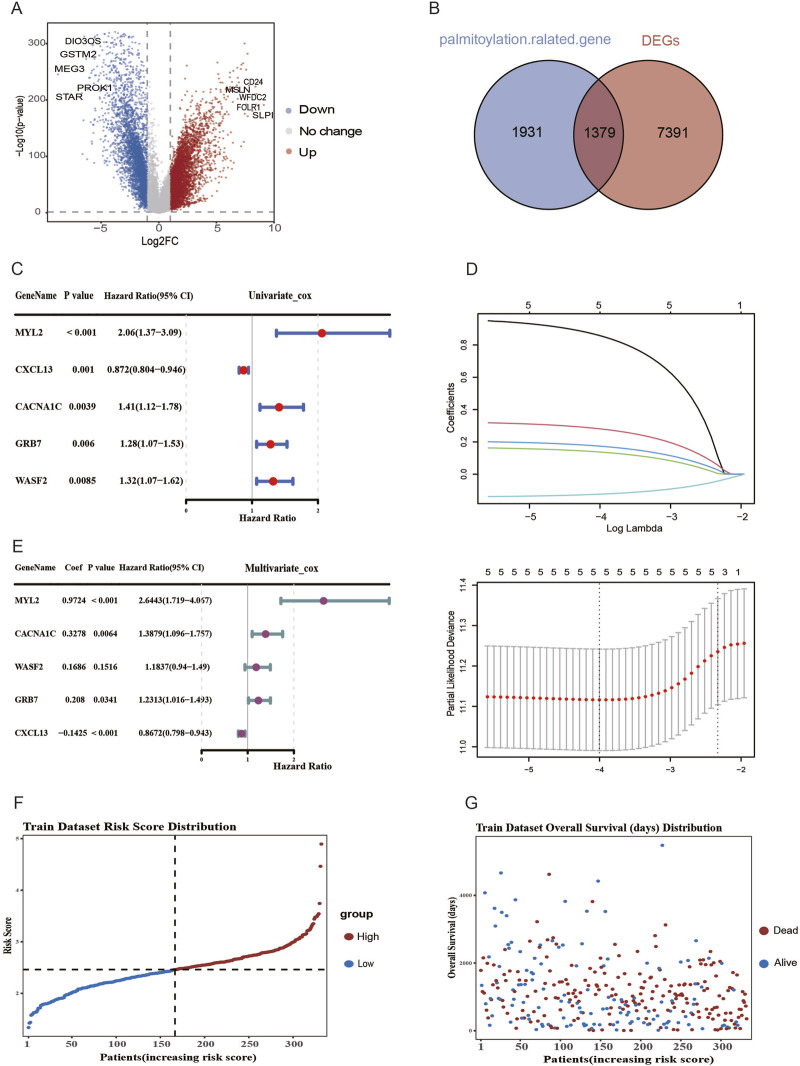
Identification of palmitoylation-related genes in ovarian cancer. **(A)** Volcano plot of differentially expressed genes between normal ovarian and tumor tissues. **(B)** Venn diagram shows the intersection of differentially expressed genes and palmitoylation-related genes. **(C)** The forest plot presents 5 palmitoylation-related genes with significant survival associations in ovarian cancers. **(D)** The results of Lasso Cox regression analysis of 5 palmitoylation-related genes. **(E)** Forest plot of multivariate Cox regression results for five palmitoylation-related genes **(F)** Distribution of risk scores in TCGA training set samples. **(G)** Distribution of survival information in TCGA training set samples.

To evaluate the expression levels of palmitoylation-related genes, we utilized RNA-seq data from the TCGA database, comparing adjacent normal ovarian tissues with ovarian cancer tissues. As illustrated in Figure 1A, differential analysis using normal cases from the GTEx project as controls revealed that 8,770 genes were significantly differentially expressed, with 4,934 upregulated and 3,886 downregulated genes. The intersection of these differentially expressed genes with 3,310 palmitoylation-related genes resulted in a set of 1,379 genes (Figure 1B). The TCGA dataset was randomly partitioned into a training set and an internal validation set at an 8:2 ratio. Utilising clinical data from ovarian cancer patients, we conducted univariate Cox regression analysis on differentially expressed palmitoylation-related protein-coding genes (Figure 1C), thereby identifying five genes that exhibited a significant association with patient survival (p < 0.01). LASSO-penalized Cox regression analysis was employed to refine candidate genes for the development of a prognostic model. The penalty parameter (λ) was determined via ten-fold cross-validation based on the minimum criterion, thereby identifying five genes (MYL2, CACNA1C, WASF2, GRB7, CXCL13) for constructing the prognostic signature (Figure 1D). Multivariate Cox regression was applied in order to calculate the β coefficient for each gene, which was then used to construct the risk score (Figure 1E). The calculation of risk scores was performed for each sample of ovarian cancer from the TCGA. In the TCGA training set, patients were dichotomised into high-risk and low-risk groups based on the median risk score. The risk score distribution exhibited slight right skewness (skewness = 0.77), with a median of 2.46, mean of 2.46, and range of 1.34–4.89 (Figure 1F). As illustrated in Figure 1G, the survival outcomes of high- and low-risk groups in the training cohort are presented.

### 3.2 Validation of the prognostic model for palmitoylation-related genes

**FIGURE 2:**
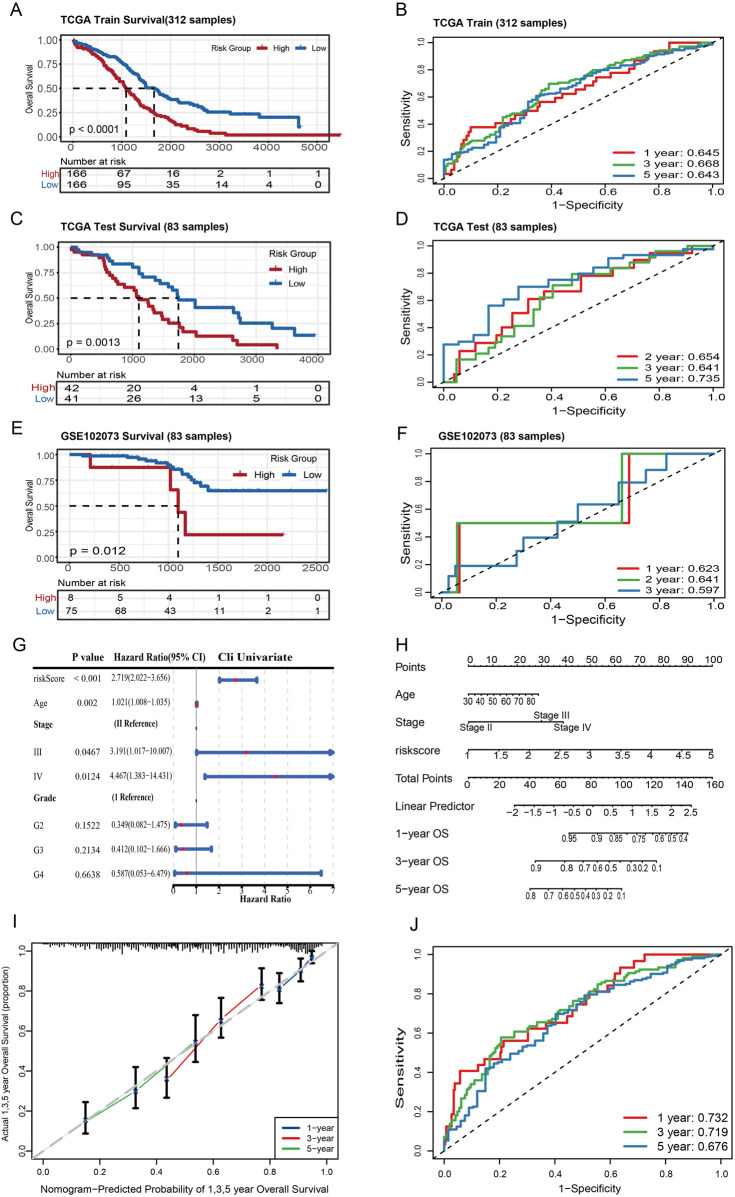
Prognostic model validation of palmitoylation-related genes. **(A,C,E)** Kaplan–Meier survival curves for high- and low-risk groups in the TCGA training, TCGA internal validation and GSE102073 cohorts, respectively, with p-values less than 0.05. **(B,D,F)** Time-dependent ROC curve analysis for predicting 1-, 3-, and 5-year survival n the TCGA training, TCGA internal validation and GSE102073 cohorts. **(G)** The forest plot showing the results of univariate Cox regression analyses of the clinical characteristics in the TCGA training cohort. **(H)** The Nomogram of the clinical characteristics in the TCGA training cohort. **(I)** The correction curve of the Nomogram. **(J)** Time-dependent ROC curve analysis of the clinical nomogram for predicting 1-, 3-, and 5-year survival in the TCGA training cohort.

We computed a risk score for each TCGA ovarian cancer sample and analyzed its correlation with patient survival within the TCGA cohort. Kaplan-Meier survival analysis confirmed the prognostic significance of the risk groups, which were categorized into high-risk and low-risk based on the median risk score. The high-risk group exhibited a poorer prognosis (Figure 2A). The area under the curve (AUC) values for the prognostic model at 1-, 3- and 5-year overall survival (OS) were 0.645, 0.668 and 0.643, respectively, confirming the robustness of the signature (Figure 2B). In order to evaluate the robustness of the prognostic model, validation was performed on the TCGA internal validation set and the GSE102073 dataset. Risk scores were calculated using the same formula derived from the training set, and patients were stratified into low- and high-risk groups using the median risk score from the training set (2.46). In the TCGA internal validation set, the area under the curve (AUC) values for the prognostic model at 1-, 3- and 5-year overall survival (OS) were 0.654, 0.641 and 0.735, respectively (Figures 2C,D). The GSE102073 dataset from the GEO database was regarded as an independent validation cohort (n = 85; 83 samples with overall survival information). Kaplan-Meier analysis revealed significantly better overall survival in the low-risk group (Figure 2E). In this external cohort, the 1-, 2- and 3-year AUCs were 0.623, 0.641 and 0.597, respectively (Figure 2F), demonstrating that the risk-score model retains reliable discriminative accuracy and robustness in an independent population.

Furthermore, univariate Cox regression analysis was performed using clinical data from the TCGA cohort (Figure 2G). Variables with p < 0.05 were selected and subjected to multivariate Cox regression analysis, which demonstrated that the risk score was independently associated with patient prognosis (Supplementary Figure S2A). In order to predict survival probabilities at 1, 3, and 5 years in a direct and efficient manner, a nomogram was constructed that integrates the risk score and clinicopathological factors (Figure 2H). The accuracy of the nomogram was validated using calibration curves (Figure 2I), and the C-index was calculated as 0.673 (95% CI: 0.59–0.75). The model was evaluated using ROC curves (Figure 2J), yielding area under the curve (AUC) values of 0.732 at 1 year, 0.719 at 3 years, and 0.679 at 5 years, indicating robust predictive performance. In order to further validate the generalisability of the model, an evaluation was performed using the TCGA internal validation set. This showed a C-index of 0.645 (95% CI: 0.489–0.801) and 1-, 3-, and 5-year AUC values of 0.654, 0.641, and 0.735, respectively (Supplementary Figures S2B,C). Despite a slight decrease in predictive performance in comparison to the training set, the validation results indicated the capacity to reliably estimate survival probabilities to a substantial degree. Collectively, the prognostic model based on palmitoylation-related genes developed in this study can serve as an independent prognostic factor for ovarian cancer patients and holds potential value for clinical risk assessment.

### 3.3 Functional characterization and mutation landscape of the model genes

**FIGURE 3:**
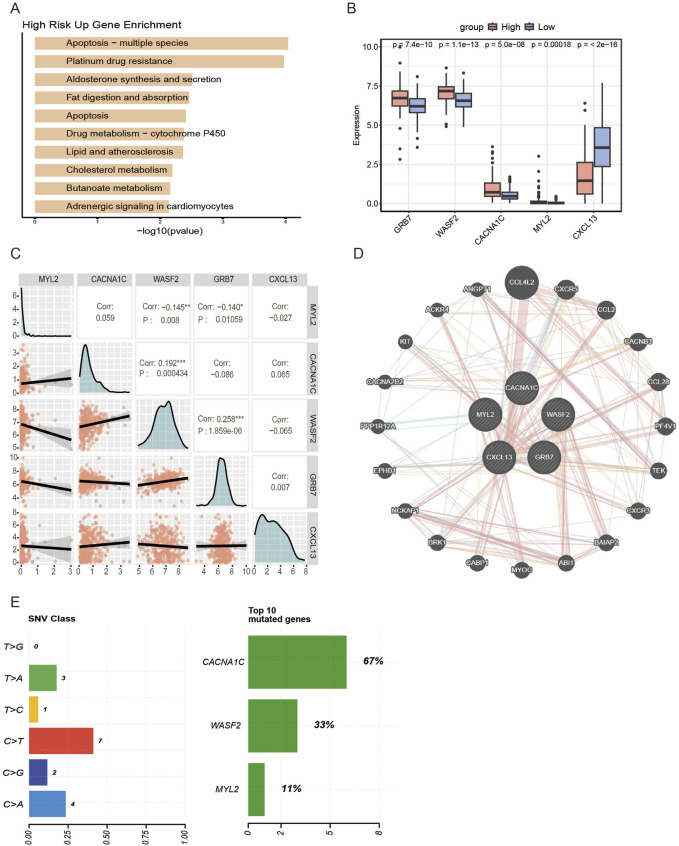
Model gene correlation analysis. **(A)** KEGG pathway enrichment analysis of upregulated genes in the high-risk group. **(B)** Boxplots show the expression differences of the model genes in the high and low risk groups in TCGA cohort. Statistical comparisons are performed using the Mann–Whitney U test. **(C)** Correlation analysis between model genes expression. **(D)** The model gene co-expression network is constructed by GeneMANIA. **(E)** Proportion and SNV type of model gene mutations.

Functional enrichment analysis was performed on differentially expressed genes between high-risk and low-risk groups in the TCGA training set. The results revealed that the high-risk group exhibited enhanced activity in pathways such as Platinum drug resistance and Lipid and atherosclerosis (Figure 3A), suggesting that drug resistance and lipid metabolism reprogramming, which provides energy for tumor metastasis, contribute to the poorer prognosis observed in the high-risk group. To investigate the primary functional mechanisms of palmitoylation-related genes in ovarian cancer, we examined the expression patterns of the five model genes in both risk groups. We observed that, except for CXCL13, the remaining four genes were significantly upregulated in the high-risk group (Figure 3B). Further correlation analysis demonstrated that WASF2 was significantly negatively correlated with MYL2 (Pearson correlation coefficient r = −0.145, p = 0.0083) and significantly positively correlated with CACNA1C (r = 0.192, p = 0.000434) and GRB7 (r = 0.258, p = 1.859e-06), while GRB7 showed a significant negative correlation with MYL2 (r = −0.14, p = 0.01059) (Figure 3C). A co-expression network of the model genes was constructed using the GeneMANIA database (Figure 3D).

We also examined the mutation status of six model genes in ovarian cancer samples. Among these, CACNA1C mutations represented the largest proportion, all of which were mistranslated mutations, primarily involving cytosine to thymidine transitions. No mutations were detected in GRB7, CXCL13 (Figure 3E). CACNA1C is responsible for encoding the α1C subunit of the voltage-dependent L-type calcium channel, which is involved in the process of Ca^2+^ influx (Wang et al., 2015). It has been demonstrated that mutations in this gene are capable of disrupting calcium channel function in cardiomyocytes. This, in turn, has been shown to cause abnormal cellular excitability and to lead to neurological and cardiovascular disorders (Herold et al., 2023). Recent studies have identified further instances of its upregulated expression in a range of tumour types, including brain tumours, leukaemia and breast cancer. In these cases, the protein regulates cell cycle progression and transcription factor activity via calcium influx, thereby modulating cell proliferation (Chang and Dong, 2021). Consequently, missense mutations in CACNA1C may promote aberrant proliferation and enhance the metastatic potential of malignant cells.

### 3.4 Analysis of immune infiltration and immunotherapy

**FIGURE 4:**
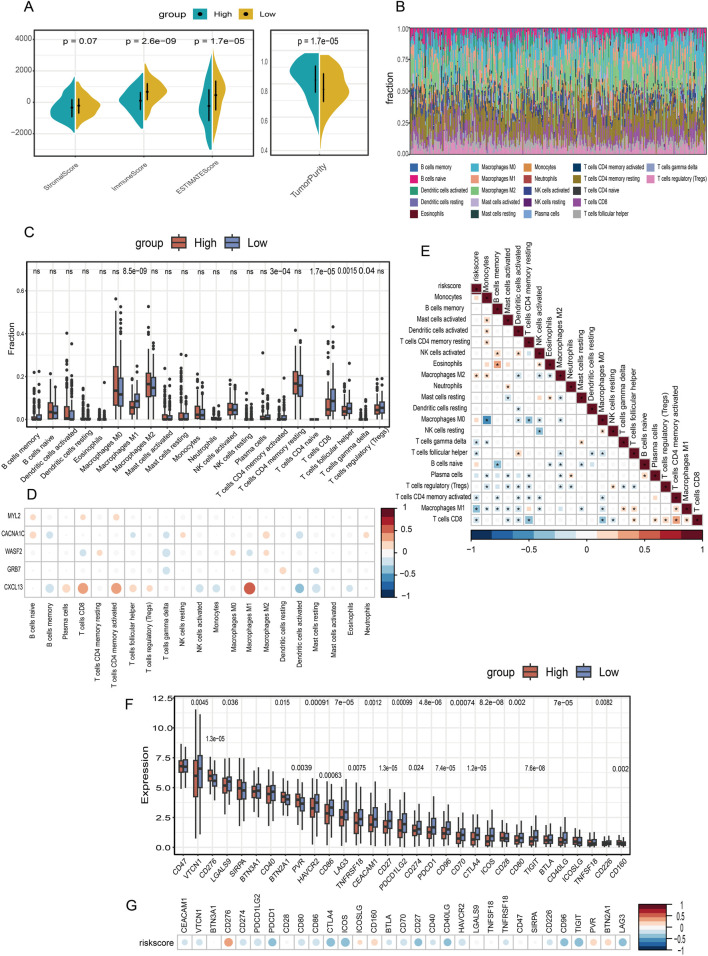
Analysis of immune infiltration and immunotherapy. **(A)** The immune score and tumor purity of samples are calculated by the ESTIMATE algorithm in TCGA training cohort. Statistical comparisons are performed using the Mann–Whitney U test. **(B)** The degree of immune infiltration is evaluated by CIBERSORT algorithm in TCGA cohort. **(C)** The infiltration of immune cells is compared between the high and low risk groups. Statistical significance was assessed using the Mann–Whitney U test and adjusted for multiple comparisons with the Benjamini–Hochberg (BH) method. **(D)** Correlation analysis between immune cell infiltration and model genes, risk score **(E)**. **(F)** The expression of 31 immune checkpoints was compared between high and low risk groups in TCGA cohort. Statistical comparisons are performed using the Mann–Whitney U test. **(G)** The correlation between risk score and the expression of 31 immune checkpoints.

Immune infiltration analysis was performed on the TCGA training cohort using CIBERSORT and ESTIMATE algorithms to evaluate immune scores. Notable differences in immune cell composition and function were observed between high-risk and low-risk groups. The high-risk group displayed significantly lower immune scores (Figure 4A) and elevated tumor purity (Figure 4B). CIBERSORT profiling revealed significant enrichment of M1 macrophages, CD8 T cells, and T follicular helper (Tfh) cells in the low-risk group (Figure 4C), a pattern validated in the TCGA internal validation set and the independent GSE165808 cohort (Supplementary Figures S3A,B). CD8 T cells, as the primary cytotoxic effectors in anti-tumor immunity, mediate direct tumor elimination; their reduction impairs this critical function (Ping et al., 2024). Insufficient CD8 T cell infiltration has been strongly associated with poor prognosis and immunotherapy resistance in ovarian cancer (Ghisoni et al., 2024). Additionally, M1-type tumor-associated macrophages (M1 TAMs) were markedly reduced in the high-risk group. M1 TAMs exhibit anti-tumor properties by promoting inflammatory responses and direct tumor cell killing. Recent studies have shown that TNF-α released by M1 macrophages enhances ovarian cancer metastatic potential through NF-κB pathway activation. The decline in M1 TAMs attenuates pro-inflammatory signaling and innate immune attack against tumors, and has been correlated with platinum resistance, disease recurrence, and poor outcomes (Cho et al., 2018). Tfh cells were also diminished in the high-risk group; these cells support B cell differentiation and anti-tumor antibody production within tertiary lymphoid structures (TLS), and their reduction compromises B and T cell helper functions while potentially indicating impaired TLS formation (Ninomiya et al., 2025). Consequently, the immune profile of the high-risk group establishes a microenvironment characterized by impaired antigen presentation, inadequate effector immune responses, and dominant immunosuppression. This milieu not only facilitates tumor immune evasion and progression but also potentially confers resistance to chemotherapy and immunotherapy, ultimately resulting in poor prognosis. These findings identify potential targets for immune-based interventions in high-risk patients. Furthermore, we examined the associations between model gene expression and immune infiltration. Notably, CXCL13 exhibited significant positive correlations with these immune cell subsets, whereas WASF2 showed significant negative correlations with them (Figure 4D). The calculated risk score was also significantly negatively correlated with CD8 T cells and M1 TAMs (Figure 4E).

Furthermore, analysis of 31 common immune checkpoint genes revealed significant differential expression between high-risk and low-risk groups (Figure 4F). The low-risk group exhibited higher expression levels of PD-1 and CD27, which represent a compensatory braking mechanism for immune activation due to increased T cell infiltration in these patients. Correlation analyses demonstrated significant inverse correlations between risk scores and the expression of most immune checkpoints (Figure 4G).

### 3.5 Single-cell expression analysis of model genes

**FIGURE 5:**
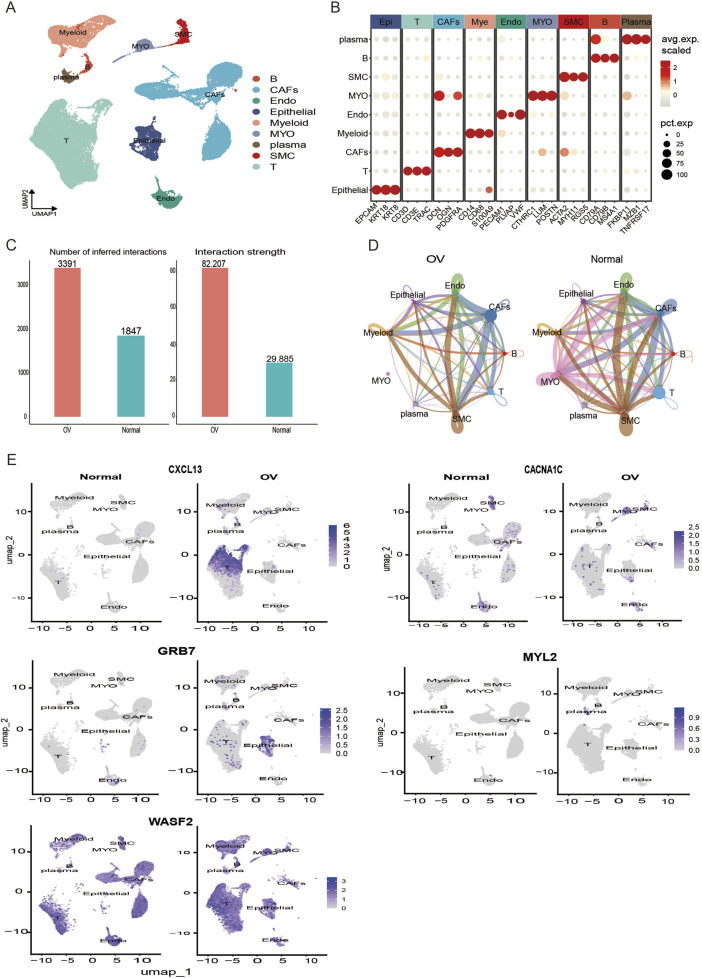
Single-cell expression analysis of model genes. **(A)** UMAP dimensionality reduction was employed to visualise cell distributions, with different colours representing distinct cell types. **(B)** Heat maps of markers that define 9 types of immune cells. **(C)** Overall cell-cell interaction counts (left) and interaction strengths (right) in ovarian cancer and normal samples. **(D)** Cell-cell interaction patterns in tumor and normal groups. **(E)** Distribution of model genes expression in different cell populations between tumor tissues and normal tissue in ovarian cancer.

The analyses presented above suggest potential differences in immune evasion mechanisms between high-risk and low-risk populations. Consequently, we proceeded to investigate model gene expression at the single-cell level. The GSE184880 dataset was obtained from the GEO database. Following rigorous quality control measures (Supplementary Figures S4A–D), 38,605 high-quality cells were retained for further analysis. The cell types were annotated using canonical markers (Che et al., 2021; Xu et al., 2025), which enabled the identification of nine distinct cell populations (Figures 5A,B), include epithelial cells (EPCAM, KRT8, KRT18), T cells (CD3D, CD3E, TRAC), cancer-associated fibroblasts (PDGFRA, DCN, OGN), myeloid cells (CD14, CD68, S100A9), endothelial cells (VWF, PECAM1, PLVAP), myofibroblasts (POSTN, CTHRC1, LUM), smooth muscle cells (ACTA2, MYH11, RGS5), B cells (CD79A, MS4A1, CD79B), and plasma cells (TNFRSF17, MZB1, FKBP11). A detailed analysis of cell-cell communication across these immune cell types revealed that both the number and strength of interactions were higher in ovarian cancer tissues compared to normal tissues (Figure 5C). T cells and cancer-associated fibroblasts (CAFs) played pivotal roles in the ovarian cancer microenvironment (Figure 5D). The subsequent stage of the research involved a comparison of the abundance of immune cell populations between normal ovarian and ovarian cancer tissues. T cells and B cells exhibited increased expression in ovarian cancer tissues, while the number of CAFs was significantly reduced (Supplementary Figure S4E). Furthermore, the expression of model genes was analysed at the single-cell level in both tissue types (Figure 5E). It is noteworthy that the protein, known as CXCR3, was predominantly localised to T cells and exhibited elevated levels of expression in tumour tissue T cells. GRB7 was predominantly expressed in epithelial cells within tumour tissues, while MYL2 expression was increased in plasma cells. Furthermore, CACNA1C expression was found to be significantly increased in myofibroblasts. This analysis provides detailed information regarding the distribution of model genes across cell types, highlighting potential downregulation in specific cell populations during disease progression.

## 4 Discussion

Ovarian cancer (OV) is one of the most lethal gynecologic malignancies, primarily due to late-stage diagnosis and limited treatment options. Despite advancements in surgical techniques and chemotherapy, the prognosis remains poor, particularly for patients with advanced disease. Consequently, many researchers have developed prognostic models for ovarian cancer based on gene expression through various bioinformatics analyses, which have demonstrated strong predictive performance. However, the prognostic model of OV palmitoylation-related genes has not been insufficiently explored. Protein palmitoylation is a reversible lipid modification that is closely related to many tumorigenesis and tumor progression (Liu et al., 2020; Shahid et al., 2020; Yan et al., 2013). For example, GBM cells have yet to enhance post-translational modifications, such as palmitoylation, which play an important role in tumor cell function (Tang et al., 2022). Recent studies have shown that palmitylation can lead to tumor suppression of T-cell immunity and impair the efficacy of immunotherapy (Rebecca et al., 2019; Du et al., 2021). However, only a few selective inhibitors targeting palmitylation proteins have been developed so far and palmitation-related proteins provide a new direction for the treatment of cancer.

In this study, we developed and validated a prognostic model based on genes involved in palmitoylation (Supplementary Figure S1). The model includes five palmitoylation-related genes (MYL2, CACNA1C, WASF2, GRB7, CXCL13). GRB7 is an adaptor protein that typically binds to kinases such as ERBB2 via its SH2 domain, thereby initiating signaling pathways for cell proliferation and survival. This protein is upregulated in various cancers, including ovarian cancer, and is regarded as a potential prognostic biomarker (Wen et al., 2024). In ovarian cancer, GRB7 expression positively correlates with M2 macrophage infiltration, and its knockdown enhances sensitivity to T-cell-mediated cytotoxicity, suggesting its role in immune evasion. Additionally, GRB7 can participate in regulating tumor progression through the ubiquitin-proteasome pathway (Wu et al., 2025). Recent research has found that ZDHHC23-mediated palmitoylation promotes protein ubiquitination and degradation. Based on this, we speculate that in ovarian cancer, GRB7 may undergo ZDHHC23-mediated palmitoylation, thereby regulating its ubiquitination and degradation process, subsequently affecting GRB7-mediated signaling pathways, tumor progression, and immune evasion mechanisms (Jeong et al., 2023). WASF2 is a pivotal protein that regulates the reorganization of the actin cytoskeleton and promotes the migration and invasion of tumour cells during metastasis. In the context of ovarian cancer, elevated WASF2 expression has been observed to be associated with tumour proliferation, platinum resistance, and a poor prognosis (Wang et al., 2023). It is noteworthy that WASF2 function is subject to regulation by post-translational modifications; for instance, zDHHC17-mediated palmitoylation has been demonstrated to impact its activity (Lemonidis et al., 2017). We speculate that in ovarian cancer, palmitoylation may further stabilise or activate WASF2, thereby enhancing its ability to drive cell motility and invasion. MYL2 encodes myosin regulatory light chain, a key regulator of smooth muscle and cardiac muscle contraction. Recent studies have demonstrated that the knockdown of MYL2 contributes to tumour progression by modulating cell proliferation and migration (Liu et al., 2025). Furthermore, research has indicated that the anchoring of myosin light chains to the plasma membrane may be regulated by palmitoylation modification (Polonais et al., 2011). CXCL13, a member of the CXC chemokine family, promotes tertiary lymphoid structure (TLS) formation in ovarian cancer, thereby coordinating cellular and humoral immunity to enhance anti-tumor responses. CXCL13 is primarily secreted by CD4 T cells, and key signaling molecules associated with T cell activation largely depend on S-palmitoylation for stable expression and plasma membrane localization to ensure proper immune signal transduction (Hundt et al., 2009). Therefore, CXCL13 expression or function is likely directly or indirectly regulated by the palmitoylation modification network, consequently influencing the shaping of the tumor immune microenvironment and potentially participating in the establishment of immune escape mechanisms.

One of the important findings of this study was the distinct pattern of immune infiltration observed between the high-risk and low-risk groups. Low-risk patients exhibited higher immune scores, increased infiltration of cytotoxic T cells, and elevated expression of immune checkpoint molecules such as PD-1 and CD27, suggesting more active immune surveillance in these individuals (Jeong et al., 2023). This observation aligns with the growing evidence that the tumor immune microenvironment plays a crucial role in determining tumor behavior and patient prognosis. Given these results, low-risk patients may benefit from immune checkpoint inhibitors, as their tumors may be more responsive to immunotherapy.

Single-cell analysis revealed that the chemokine, CXCL13, was primarily localized to T cells and exhibited higher expression in T cells within tumour tissues. GRB7 was predominantly expressed in epithelial cells of tumour tissues, while MYL2 expression was increased in plasma cells and CACNA1C expression was significantly upregulated in myofibroblasts. The altered expression patterns of model genes across immune cell populations may imply a potential mechanism whereby palmitoylation-related pathways modulate immune cell activities within the tumour microenvironment, thereby promoting cancer progression and immune evasion.

A drug sensitivity analysis was conducted utilising the GDSC database, which resulted in the identification of 278 chemotherapy drugs that exhibited significant differential sensitivity between high-risk and low-risk groups. The high-risk group demonstrated significantly elevated IC50 values for the majority of drugs, suggesting an augmented degree of drug resistance (Supplementary Figure S5A). We performed correlation analyses between model genes and a panel of 10 chemotherapeutic agents—including paclitaxel and docetaxel (Hashemi et al., 2023), which are widely used in clinical oncology practice (Hundt et al., 2009). Our results demonstrated that CACNA1C and WASF2 were significantly and positively correlated with resistance to multiple drugs (Yang et al., 2022) (Supplementary Figure S5B). Additionally, The prediction of targeted drugs for differentially expressed genes between risk groups was conducted using the CMAP database (Subramanian et al., 2017) (Supplementary Figure S5C). The top three therapeutic candidates were analysed and subjected to molecular docking against model genes. The results indicated that biperiden could potentially target CACNA1C, fluoxetine could target WASF2, and FIT could target CXCL13 (Supplementary Figures S5E,F). This analysis may provide valuable insights for chemotherapeutic strategies in ovarian cancers with distinct molecular heterogeneity. It is noteworthy that the aforementioned predictions constitute exploratory findings,the feasibility of both therapeutic targets and drugs remains to be rigorously validated through experimental studies.

From a clinical perspective, our findings indicate that genes related to palmitoylation can serve as valuable biomarkers for stratifying ovarian cancer risk. The capacity of these genes to predict survival outcomes and immune responses highlights their potential as prognostic tools. Moreover, due to their association with immune infiltration, targeting palmitoylation pathways in conjunction with immunotherapy may represent a promising therapeutic strategy, particularly for low-risk patients exhibiting heightened immune activity.

Notwithstanding the study’s merits, it is imperative to acknowledge its limitations, which are as follows. Firstly, ovarian cancer and normal tissue samples were sourced from TCGA and GTEx, respectively. Variations in the use of sequencing platforms, experimental workflows, and sample handling between these databases are susceptible to the induction of batch effects. However, given the absence of normal tissue samples in the TCGA database, the implementation of batch effect correction based on data source would inevitably result in the elimination of inherent biological variations between cancerous and normal specimens. Consequently, an exploratory approach was adopted, with these differences being retained in the present study. In addition (Ni et al., 2025), our analysis relied on publicly available data sets that may not fully reflect the heterogeneity of ovarian cancer in different populations, which could introduce bias in the findings. Although the results are promising, validation in a larger and more representative cohort will be essential to confirm the robustness and generalizations of the prognostic model. Furthermore, although significant associations were observed between palmitoylation-related genes and immune infiltration, these findings are relevant and further mechanistic studies are needed to elucidate how these genes specifically regulate immune cell behavior and contribute to genomic instability in ovarian cancer. Experimental validation of these associations is essential to fully understand the underlying biological mechanisms. Future studies should prioritize validating this prognostic model in prospective clinical trials and exploring the role of palmitylation in modulating the immune response in ovarian cancer. These mechanistic insights could reveal novel therapeutic strategies to target these pathways and improve patient outcomes.

## Data Availability

The original contributions presented in the study are included in the article/Supplementary Material, further inquiries can be directed to the corresponding authors. Analysis code are publicly available at https://github.com/LiuTD123/CRCpalmitoylation/.
