# Supplementary material for: A novel prognostic model for ovarian cancer: construction, validation, and therapeutic insights
Source: Front Genet. 2026 Apr 22;17:1603788. doi: 10.3389/fgene.2026.1603788 (PMC13143230; doi:10.3389/fgene.2026.1603788)
Supplement: Supplementary file 1 [file DataSheet1.docx]

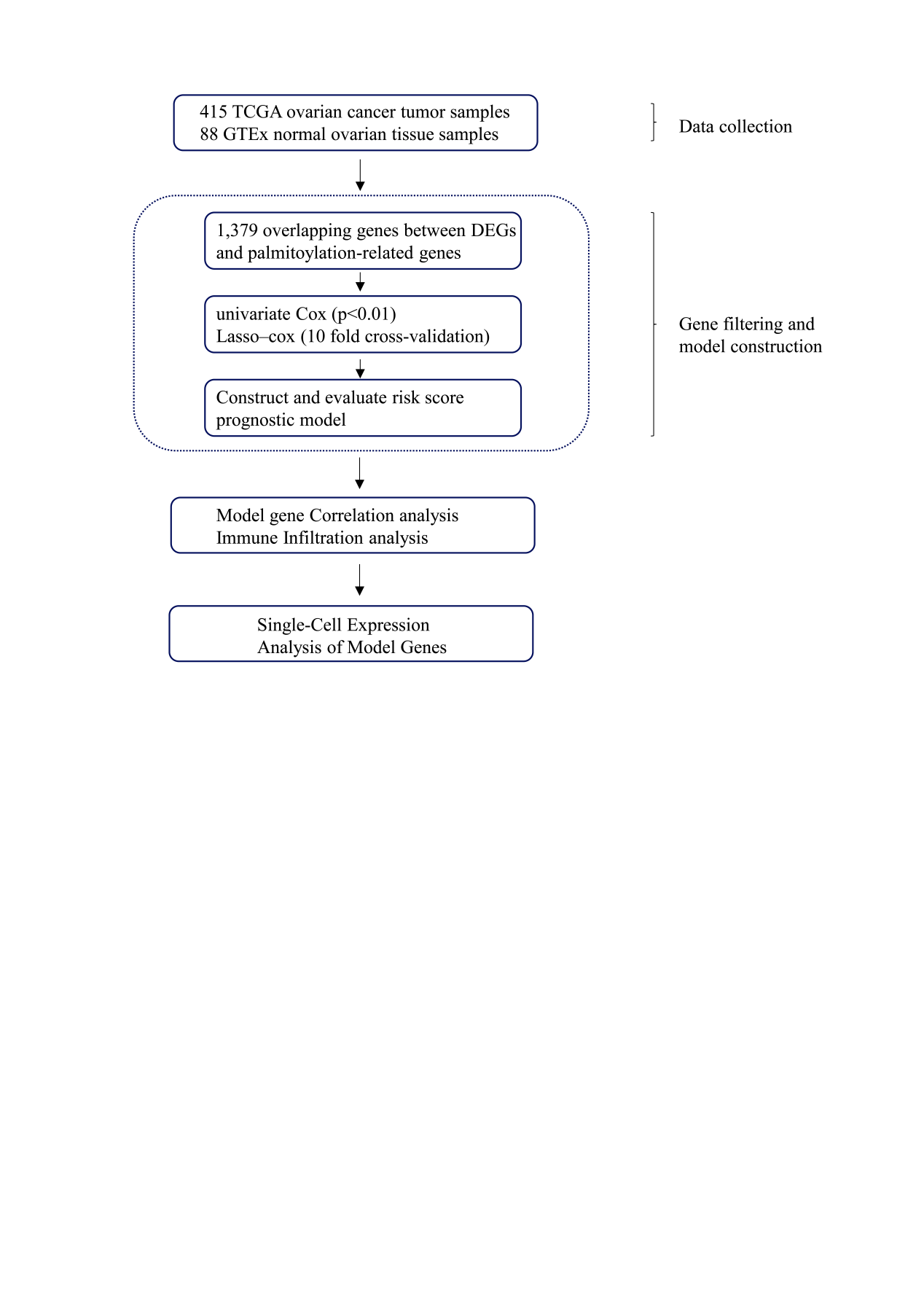


Supplement Figure1. Research analysis workflow.


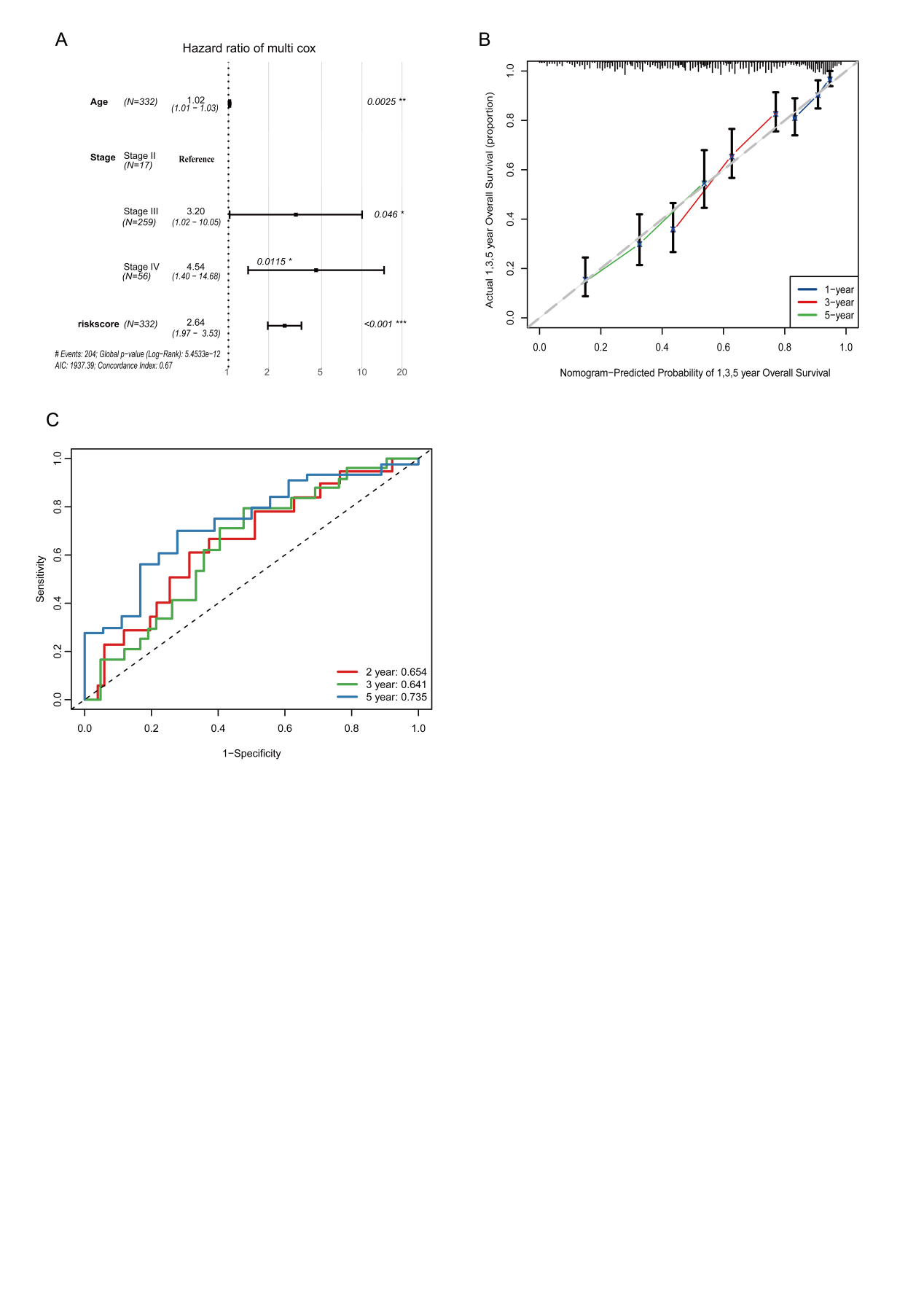


Supplement Figure2. A.Forest plot of multivariate Cox regression results for the clinical characteristics in the TCGA training cohort. B. The correction curve of the TCGA internal validation cohort. C.Time-dependent ROC curve analysis of the clinical nomogram for predicting 1-, 3-, and 5-year survival in the TCGA internal validation cohort

Supplement Figure3. A.The infiltration of immune cells is compared between the high and low risk groups in TCGA internal validation cohort. Statistical significance was assessed using the Mann–Whitney U test and adjusted for multiple comparisons with the Benjamini–Hochberg (BH) method. B.The infiltration of immune cells is compared between the high and low risk groups in GSE165808 cohort. Statistical significance was assessed using the Mann–Whitney U test and adjusted for multiple comparisons with the Benjamini–Hochberg (BH) method.


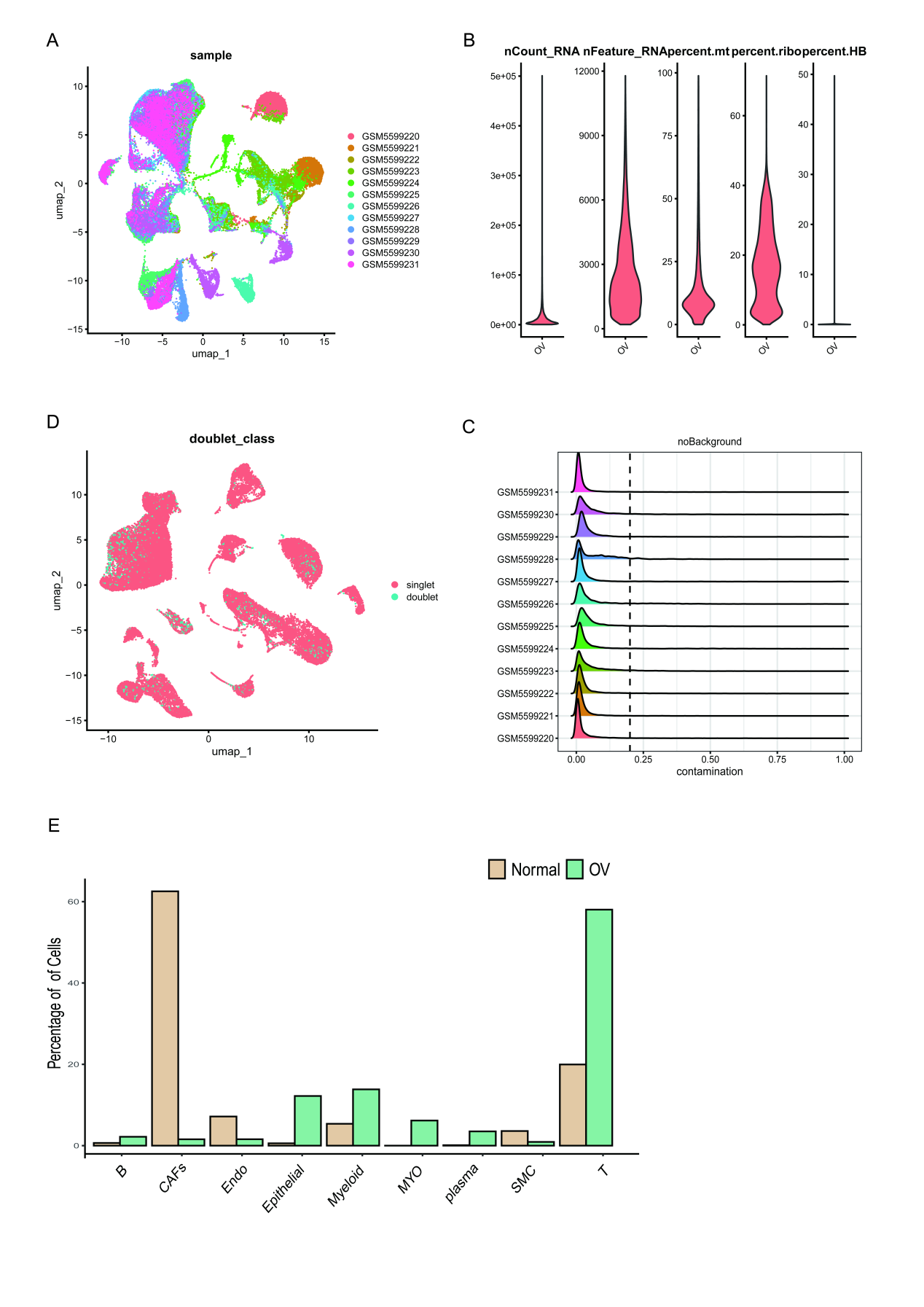


Supplement Figure4. A. Batch effects in single-cell data. B. Initial quality control metrics for single-cell data, including total UMI counts, number of features detected per cell, mitochondrial gene percentage, ribosomal gene percentage, and proportion of red blood cell expression. C. Contamination estimation for each sample using decontX. D. Doublet prediction using scDblFinder. E. Proportions of cell types in ovarian cancer and normal samples


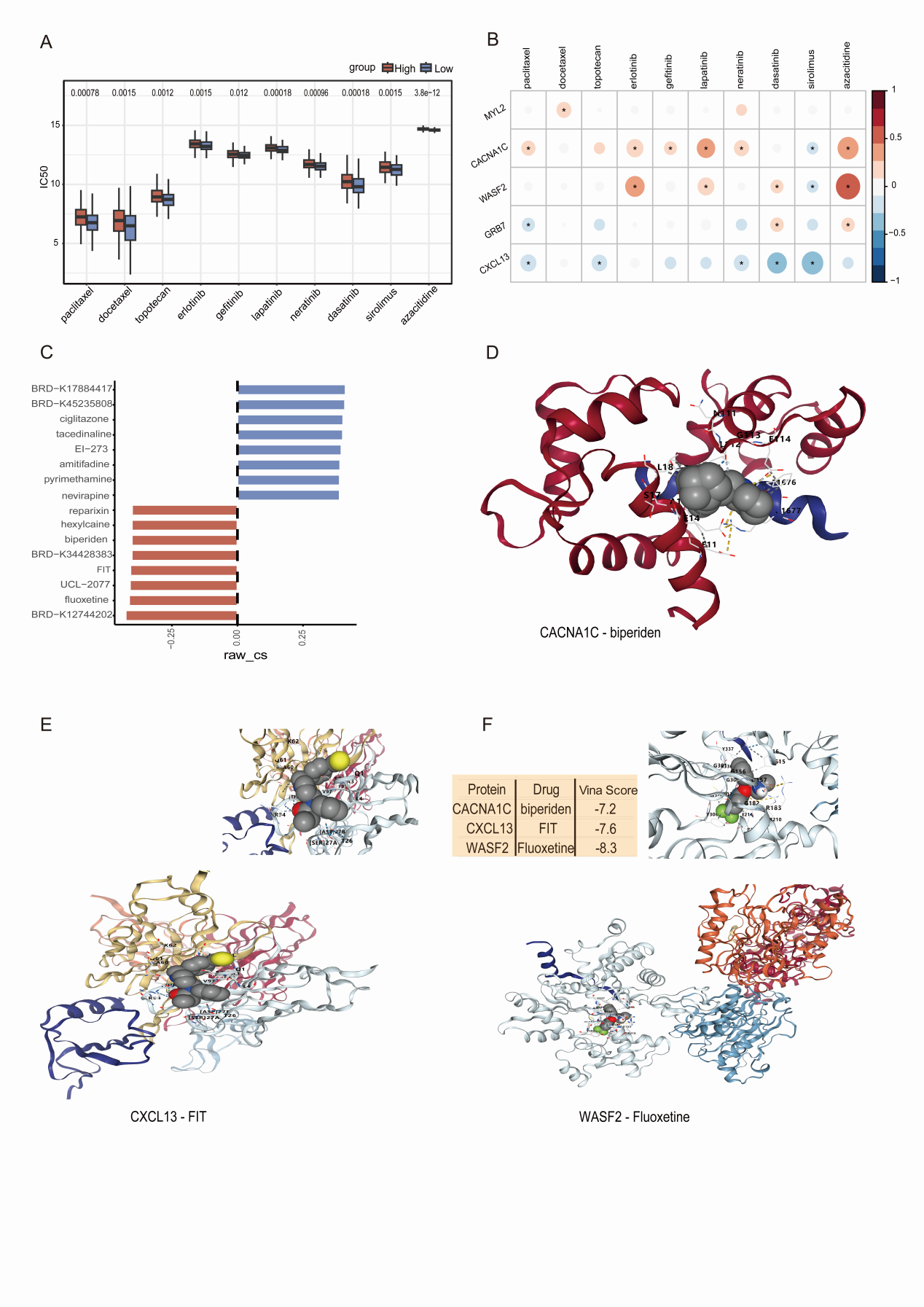


Supplement Figure5 Drug Susceptibility Analysis. A. Drug sensitivity is compared between the high and low risk groups. Boxplots showing the 10 drugs with the significant differences. Statistical comparisons are performed using the Mann–Whitney U test.B. Correlation between the IC50 values of ten drugs and the model genes. C. The CMAP database is used to predict the targeted drugs for high-risk populations. D-F. The top three predicted targeted drugs with the most therapeutic potential were docked with the model genes.
